# Chronic heavy alcohol consumption influences the association between genetic variants of *GCK* or *INSR* and the development of diabetes in men: A 12-year follow-up study

**DOI:** 10.1038/s41598-019-56011-y

**Published:** 2019-12-27

**Authors:** Han Byul Jang, Min Jin Go, Sang Ick Park, Hye-Ja Lee, Seong Beom Cho

**Affiliations:** 1 Center for Biomedical Science, Korea National Institute of Health, Cheongju, Chungcheongbuk-do Republic of Korea; 2 Center for Genome Science, Korea National Institute of Health, Cheongju, Chungcheongbuk-do Republic of Korea

**Keywords:** Medical genetics, Type 2 diabetes

## Abstract

Chronic heavy alcohol consumption is a risk factor for diabetes, which is characterized by impaired β-cell function and insulin resistance. We aimed to determine whether the longitudinal associations between genetic variants of glucokinase (*GCK*) and insulin receptor (*INSR*) and the risk of developing diabetes were influenced by chronic heavy alcohol consumption. Data were obtained from the Korean Genome and Epidemiology Study. To identify candidate variants, 1,520 subjects (726 non-drinkers and 794 heavy drinkers) were included in the baseline cross-sectional study. After excluding patients with diabetes at baseline and those with insufficient data on diabetes incidence, prospective analyses were conducted in 773 subjects (353 non-drinkers and 420 heavy drinkers). In the baseline cross-sectional study, one SNP (rs758989) in *GCK* and four SNPs (rs7245757, rs1035942, rs1035940, and rs2042901) in *INSR* were selected as candidate SNPs that interact with alcohol to affect prediabetes and diabetes. We identified that these *GCK* and *INSR* polymorphisms are affected by chronic heavy alcohol consumption and have an effect on the incidence of diabetes. The incidence of diabetes was increased in chronic heavy alcohol drinkers carrying the *C* allele of *GCK* compared with never-drinkers with the C allele (HR, 2.15; 95% CI 1.30–3.57), and was increased in chronic heavy alcohol drinkers who were not carrying the *INSR* haplotype (−/−) compared with never-drinkers carrying the AACT haplotype (HR, 1.98; 95% CI 1.24–3.18). Moreover, we observed that the aggravating effects on the late insulin secretion (I/G_120_ and I/G _AUC 60–120_) in individuals who were chronic heavy drinkers with C allele of *GCK*. In the *INSR* haplotype, chronic heavy drinkers not carrying AACT were associated with lower disposition index. These results potentially suggest that chronic heavy alcohol consumption induce β-cell dysfunction partially mediated by decreased *GCK* expression or decline of insulin sensitivity via inhibition of *INSR*, thereby contributing to the development of diabetes.

## Introduction

Diabetes is associated with serious comorbidities, including macrovascular diseases (hypertension, hyperlipidemia, heart attack, coronary artery disease, and stroke), microvascular diseases (retinopathy, nephropathy, and neuropathy), and cancers. Although the etiology of diabetes is complex, the key factor is chronic hyperglycemia due to impaired β-cell function and insulin sensitivity^1^. Thus, understanding the regulation of glucose homeostasis is crucial for preventing diabetes and its complications.

The genes involved in insulin actions and the glucose-stimulated insulin secretion (GSIS) pathway are assumed to contribute to the development of diabetes^2,3^. Both the insulin receptor (*INSR*) and glucokinase (*GCK*) play important roles in insulin-related pathways. The insulin signaling cascade is mediated by *INSR*, which phosphorylates the insulin receptor substrate (*IRS*) to activate the mitogen-activated protein kinase and phosphoinositide 3-kinase (PI3K) pathways and is essential for insulin actions. Mutations in the *INSR* gene affect insulin function and cause insulin resistance, which leads to the development of type 2 diabetes^4^. *GCK* also plays an important role in the regulation of glucose metabolism and insulin secretion, acting as a metabolic sensor for GSIS in pancreatic β cells. Studies have reported that chronic hyperglycemia-induced β-cell dysfunction is closely related to downregulation of *GCK* expression^5^. Any functional defect in *GCK* suppresses glucose utilization in the liver and decreases insulin secretion from β cells, which may cause diabetes.

Diabetes is influenced by the multifactorial interplay among genetic and environmental factors, including alcohol consumption. Heavy alcohol consumption is a risk factor for diabetes, which is characterized by impaired insulin secretion and insulin resistance^6,7^. Thus, we questioned whether the associations between glucose homeostasis-related gene mutations and diabetes were modified by alcohol consumption. To our knowledge, no study has assessed the influence of chronic heavy alcohol consumption on the relationships between genetic variants and the incidence of diabetes. Therefore, we evaluated the effects of the interactions between *GCK* and *INSR* single-nucleotide polymorphisms (SNPs) and chronic heavy alcohol consumption on β-cell function, insulin sensitivity, and development of diabetes in a 12-year follow-up cohort study.

## Materials and Methods

### Study design and population

Data were obtained from the Ansan–Ansung cohort study conducted by the Korea National Institute of Health as part of the Korean Genome and Epidemiology Study. A total of 8,840 participants (4,182 men and 4,658 women) aged 40–69 years were recruited from 2001 to 2002 and were followed up by survey every 2 years^8^. We included data from 2001 to 2014 and only those from men, because most women abstained from alcohol. The participants were asked about their current drinking status (never, former, or current) and their average monthly amount and frequency of alcohol consumption. From these measures, we derived the average alcohol consumption in units per day for each participant. Written informed consent was obtained from all participants. The study was approved by the Institutional Review Board of the National Biobank of Korea and the Korea National Institute of Health (2017-02-08-PE-A). The study procedures were carried out in accordance with approved guidelines.

### Baseline cross-sectional study

To screen the *GCK* and *INSR* genetic variants that interact with alcohol and contribute to the development of diabetes, baseline cross-sectional data was used. Participants who had insufficient OGTT data (n = 239) and those who did not respond to the alcohol consumption questionnaire (n = 128) were excluded from the cross-sectional analysis. Because there have been reports of J or U-shape associations between alcohol consumption and the diabetes incidence, we excluded from the analysis all former (n = 379) and current drinkers who reported consuming ‘less than 30 g/day’ of alcohol (n = 1,896). We also eliminated current drinkers who reported a drinking period of less than 10 years (n = 20). Finally, the data from 1,520 subjects were included in the analysis and the subjects with an average consumption of 30 g/day or more was considered to heavy alcohol drinkers (726 abstainers and 794 heavy drinkers; Supplementary Fig. S1.A). The characteristics of subjects are shown in Supplementary Table S1).

### Longitudinal study

Longitudinal data was used to test whether selected *GCK* and *INSR* genetic variances interact with long-term alcohol consumption and affect the incidence of diabetes. Participants with diabetes at baseline (n = 461) and those with insufficient data on diabetes incidence (n = 583) were excluded. The alcohol consumption questionnaire was conducted every 2 years during the follow-up period, and we classified the participants into three groups at each survey point according to their alcohol consumption patterns, as follows: no alcohol consumption, low-to-moderate consumption (<30 g/day alcohol), and high alcohol consumption (≥30 g/day alcohol). To characterize long-term alcohol consumption patterns better and to minimize intra-individual variation, we eliminated the subjects who had a response rate (number of responses) to <65% during the tracking period (n = 203). Never-drinkers were classified as those who were consistent with the response to ‘did not drink’ during the follow-up period (n = 353). When the ratio of the number of high alcohol consumption (≥30 g/day at each survey point) to the number of responses to alcohol consumption questionnaire during the tracking period exceeded 65%, we classified it as chronic heavy drinkers (n = 420). Finally, the data from 773 subjects (353 never-drinkers and 420 chronic heavy drinkers) were included in the analysis (Supplementary Figs. S1B and S2).

### Measurements of insulin parameters

Professionally trained personnel performed the anthropometric examinations and blood collections in the cohort study using a standardized protocol. Each participant underwent a 2-h 75-g oral glucose tolerance test (OGTT), both at the time of enrollment and every 2 years thereafter. Plasma samples were collected at 0 min, 1 h, and 2 h post-glucose consumption in the OGTT to measure glucose and insulin concentrations, which were measured using the hexokinase method and radioimmunoassay, respectively. Diabetes was defined as a fasting glucose level ≥126 mg/dL or 2 h post-OGTT glucose level ≥200 mg/dL. In addition, participants who reported current therapy with anti-diabetes medication or insulin administration were considered to have diabetes. Insulin sensitivity was measured with the composite insulin sensitivity index (ISI) based on the 0 min, 1 h, and 2 h glucose (mg/dL) and insulin (μU/mL) levels measured in the OGTT^9^. All other insulin secretion indices were derived from the OGTT with insulin concentration given in μU/mL and glucose concentration given mmol/L. Pancreatic β-cell function was estimated with the homeostasis model assessment of β function (HOMA-B), 1-h insulinogenic index (IGI_60_), the ratio of area under the insulin curve to area under the glucose curve from 1 h to 2-h (I/G _AUC 60–120_), and the ratio of insulin to glucose at 2 h post-OGTT (I/G_120_). The IGI was calculated as (insulin _60min_ − insulin _0min_)/(glucose _60min_ − glucose _0min_)^10,11^. Disposition index derived from OGTT was estimated as IGI_60_ x composite ISI. Smoking status was classified as current, former, or never smoker, and physical activity was calculated by multiplying the time spent engaging in activities at a particular intensity level by the metabolic equivalent of task score.

### Screening of genetic variants

Samples from the Ansan–Ansung cohort study were genotyped using the Affymetrix Genome-Wide Human SNP Array 5.0 (Affymetrix Inc., Santa Clara, CA, USA) and processed using the Bayesian robust linear model with the Mahalanobis distance classifier for genotype calling^12^. Of 3.5 million variants, 27 SNPs present in the *GCK* and *INSR* loci were available for the analysis. To screen the *GCK* and *INSR* genetic variants that interact with alcohol and contribute to the development of diabetes, a genotype-environment interaction analysis for combined prediabetes and diabetes or diabetes alone was tested with generalized logistic regression additive model with interaction terms. Finally, we selected one SNP (rs758989) in *GCK* and four SNPs (rs7245757, rs1035942, rs1035940, and rs2042901) in *INSR* as candidate genetic variants (*p* < 0.05, Supplementary Table S2).

### Statistical analysis

Statistical analyses were performed using the PLINK (ver. 1.9) and SAS software packages (ver. 9.4; SAS Institute Inc., Cary, NC, USA). Data are presented as means ± SD, numbers (%), or hazard ratios (HRs) with 95% confidence intervals (CIs). Variables with non-normal distributions were log-transformed prior to analysis. For each participant, we calculated person-years of follow-up from the year the questionnaire was completed to the year of diabetes diagnosis or censoring. The statistical significance of between- and among-group differences was assessed using general linear models adjusted for age or for age, physical activity, smoking, ALT (or AST for ß-cell function and the insulin sensitivity index), family history of diabetes, and BMI followed by Duncan’s post-hoc test (for among-group differences). The relationships of genotype and alcohol consumption with the incidence of diabetes were evaluated using Kaplan–Meier survival analysis, and the differences between survival curves were determined using the log-rank test. The combination effect of the genotype–alcohol consumption on the development of diabetes was evaluated with the Cox proportional hazards model and adjustments for age, physical activity, smoking status, family history of diabetes, ALT and BMI. All reported *p* values were two-tailed and the results were considered statistically significant at *p* < 0.05.

## Results

### General characteristics of the study population and genotype distribution

The baseline characteristics of the 773 participants (353 never-drinkers and 420 chronic heavy drinkers) according to their alcohol consumption habits during follow-up are shown in Table 1. All participants had normal glucose tolerance at baseline; 66 of the 353 never-drinkers and 110 of the 420 chronic heavy drinkers developed type 2 diabetes during the 12-year follow-up period (mean follow-up: 8.3 years). Blood pressure (SBP and DBP), liver enzyme (AST, ALT, and GTP), lipid (high-density lipoprotein–cholesterol and triglyceride), blood sugar (glucose levels at 0 min, 1 h, and 2 h post-OGTT), pancreatic β-cell function (HOMA-B, IGI_60_, I/G _AUC 60–120_, I/G_120_ and disposition index), physical activity, and smoking were higher in chronic heavy drinkers than in the never-drinkers (all *p* ≤ 0.0241). Chronic heavy drinkers had a higher rate of developing diabetes than the never-drinkers (HR, 1.41; 95% CI, 1.00–1.98; Supplementary Table S3.B).

**Table 1:** Baseline characteristics of the study participants according to alcohol consumption patterns over the follow-up period.

	Never-drinkers (N = 353)	Chronic heavy drinkers (N = 420)	*P*-value^b^
Age (y)	53.3 ± 9.3	49.6 ± 8.2	<0.0001
BMI (kg/m^2^)	23.7 ± 3.0	24.3 ± 2.9	0.1454
SBP (mmHg)	119.9 ± 15.8	123 ± 16.3	<0.0001
DBP (mmHg)	79.9 ± 10.7	83.5 ± 10.7	<0.0001
AST (IU/L)^a^	26.2 ± 17.1	33.9 ± 23.0	<0.0001
ALT (IU/L)^a^	26.8 ± 18.6	32.3 ± 21.0	0.0002
GTP (IU/L)^a^	27.9 ± 19.7	84.3 ± 118.7	<0.0001
Total cholesterol (mmol/L)	5.1 ± 0.9	5.1 ± 1.0	0.5584
HDL-cholesterol (mmol/L)	1.2 ± 0.3	1.3 ± 0.3	<0.0001
Triglycerides (mmol/L)^a^	1.6 ± 1.0	2.2 ± 1.8	<0.0001
Fasting glucose (mmol/L)	4.9 ± 0.5	5.1 ± 0.5	<0.0001
1 h glucose (mmol/L)	8.5 ± 2.5	9.1 ± 2.5	<0.0001
2 h glucose (mmol/L)	6.4 ± 1.9	6.7 ± 1.9	0.0051
Fasting insulin (μU/mL)^a^	7.0 ± 5.7	6.6 ± 3.3	0.7919
1 h insulin (μU/mL)^a^	29.4 ± 27.5	30.1 ± 30.3	0.3509
2 h insulin (μU/mL)^a^	25.0 ± 23.7	22.8 ± 24.2	0.0826
HOMA-B (%)^a^	113.7 ± 110	90.2 ± 51.5	0.0007
IGI_60_^a^	9.3 ± 15.7	8.5 ± 15.8	0.0180
I/G_AUC 60–120_^a^	3.7 ± 2.7	3.4 ± 3.0	0.0033
I/G_120_^a^	3.8 ± 3.1	3.3 ± 2.9	0.0036
Composite ISI^a^	13.1 ± 12.6	12.3 ± 9.8	0.8053
Disposition index^a^	75.7 ± 90.3	67.0 ± 90.0	0.0049
MET (Physical activity)	10223.6 ± 6309.4	10916.9 ± 6582.8	0.0033
Smoking (ex/current)	26.4%/38.6%	28.1%/61.7%	<0.0001
Family history of diabetes	9.4%	12.6%	0.1497

Data are unadjusted means (SD) or %; BMI, body mass index; SBP, systolic blood pressure; DBP, diastolic blood pressure; ALT, alanine aminotransferase; AST, aspartate aminotransferase; r-GTP, gamma glutamyltranspeptidase; HOMA-B, homeostasis model assessment-beta; IGI, insulinogenic index; I/G, the ratio of insulin to glucose; AUC, area under the curve; ISI, insulin sensitivity index; MET, metabolic equivalent of task.

^a^Log transformations before analysis.

^b^*P* value were calculated by generalized linear regression analysis with age for continuous parametric variables and Chi-square test for categorical variables.

The genotype distributions of selected *GCK* and *INSR* genetic variants were in Hardy-Weinberg equilibrium (P > 0.05). From the LD test, selected four *INSR* SNPs were found to be highly linked (*D’* ≥ 0.96, *r*^2^ ≥ 0.90). The *INSR* haplotype analysis was performed for the minor A allele of SNP rs7245757, combined with the minor alleles of rs1035942, rs1035940, and rs2042901 (*ht*: AACT). Supplementary Table S4 and S5 show the baseline characteristics of the study population according to the genotype of *GCK* rs758989 and *INSR* haplotype. Significant genotype-related difference in 2-h glucose levels was detected for *GCK* rs758989. In *INSR* haplotype, AACT haplotype carriers had lower glucose levels during the OGTT and higher 2-h insulin levels. There was no association between *GCK* genotype and the incidence of diabetes, but non-AACT haplotype had a higher diabetes incidence compared to AACT haplotype (HR, 1.45; 95% CI, 1.06–1.98; Supplementary Fig. S3).

### Effect of chronic heavy alcohol consumption and *GCK*/*INSR* variants on the incidence of diabetes

In the alcohol consumption-stratified analysis, the minor allele of *GCK* (rs758989) and the major allele of *INSR* (rs7245757, rs1035942, rs1035940, and rs2042901) had a tendency to increase the incidence of diabetes in the chronic heavy drinkers; however, there were no significant genotype-related differences in the never-drinkers (Supplementary Table S6). Because of the small sample number of *GCK* and *INSR* homozygous minor genotype carriers with the incidence of diabetes, the analysis according to genotype was performed using the dominant model for *GCK* and the haplotype model for *INSR*. The aggravating effects of the chronic heavy alcohol consumption on the incidence of diabetes were only present in the C allele of *GCK* (Table 2). There were no significant alcohol-related differences in each *INSR* haplotype groups.

**Table 2:** Associations of single-nucleotide polymorphisms in *GCK* and *INSR* with chronic heavy alcohol consumption and their effect on the incidence of diabetes.

	Genotype or Haplotype (HR, 95% CI)				*P*-value
***GCK*** **(rs758989)**					
Model 1	TT		TC + CC		
Never-drinkers	1	(reference)	0.67	(0.40–1.12)	0.1247
Chronic heavy drinkers	1	(reference)	1.46	(1.00–2.15)	0.0522
Model 2					
Never-drinkers	1	(reference)	1	(reference)	
Chronic heavy drinkers	0.90	(0.57–1.41)	2.29	(1.36–3.87)	
*P*-value	0.6304		**0**.**002**		
Model 3					
Never-drinkers	1.5	(0.89–2.49)	1	(reference)	**0**.**0029**
Chronic heavy drinkers	1.5	(0.90–2.49)	2.15	(1.30–3.57)	
***INSR*** **(4 SNPs haplotype)**					
Model 1	AACT Carrier		Non-Carrier		
Never-drinkers	1	(reference)	1.24	(0.74–2.08)	0.4115
Chronic heavy drinkers	1	(reference)	1.56	(1.05–2.31)	**0**.**0279**
Model 2					
Never-drinkers	1	(reference)	1	(reference)	
Chronic heavy drinkers	1.45	(0.86–2.45)	1.42	(0.87–2.30)	
*P*-value	0.1646		0.1616		
Model 3					
Never-drinkers	1	(reference)	1.23	(0.74–2.06)	**0**.**0044**
Chronic heavy drinkers	1.25	(0.76–2.06)	1.98	(1.24–3.18)	

*P*-values were calculated by Cox proportional hazard analysis with adjustment for age, physical activity, family history of diabetes, smoking status, BMI, and ALT.

Model 1, compared with homozygous TT genotype or AACT haplotype; Model 2, compared with never-drinker group; Model 3, compared with never-drinkers carrying the C allele or AACT haplotype.

**Figure 1:**
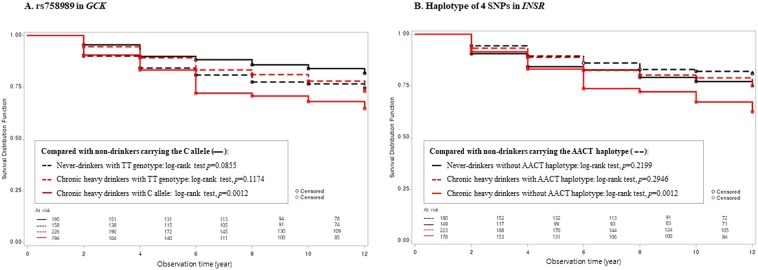
Kaplan-Meier curves for the incidence of diabetes according to the combined model of genetic variants ((**A**) *GCK* or (**B**) *INSR*) and chronic heavy alcohol consumption.

**Figure 2:**
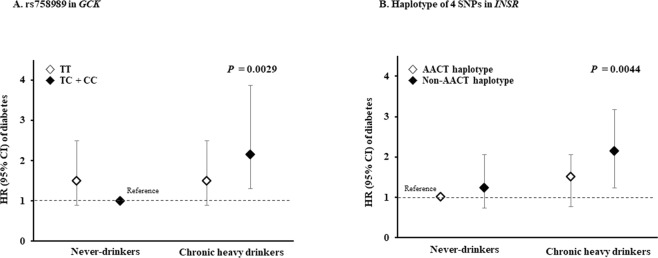
Effect of the interaction between chronic heavy alcohol consumption and genetic variants of (**A**) *GCK* or (**B**) *INSR* on diabetes incidence. *P*-values were calculated by Cox proportional hazard analysis with adjustment for age, physical activity, family history of diabetes, smoking status, and BMI.

In the combined analysis of alcohol consumption and genetic variants groups, the Kaplan–Meier curves showed that chronic heavy drinkers carrying the C allele of *GCK* rs758989 and those not carrying the AACT haplotype (−/−) of *INSR* had a higher probability of developing diabetes over the 12-year follow-up period (log-rank test *p* = 0.0012 compared to never-drinkers with C allele of *GCK* and *p* = 0.0012 compared to never-drinkers with AACT haplotype, respectively, Fig. 1). We also detected a combination effect between genetic variants of *GCK* or *INSR* and chronic heavy alcohol consumption on the incidence of diabetes (*p* = 0.0029 and 0.0044, respectively, Table 2 and Fig. 2). The *GCK* rs758989 C allele and lack of the *INSR* AACT haplotype (−/−) exhibited interactions with chronic heavy alcohol consumption in their effects on the incidence of diabetes. The HR for the incidence of diabetes was 2.15 (95% CI 1.30–3.57) for chronic heavy drinkers carrying the C allele compared with never-drinkers who were C allele at *GCK* rs758989. The HR for the incidence of diabetes was 1.98 (95% CI 1.24–3.18) for chronic heavy drinkers not carrying the AACT haplotype (−/−) of *INSR* compared with never-drinkers carrying the haplotypes (*ht*/*ht*) and (*ht*/−).

**Figure 3:**
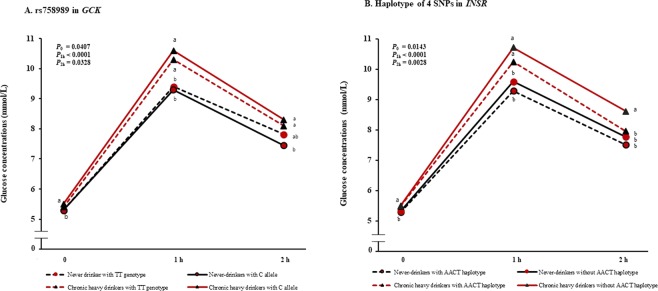
Glucose concentrations during the oral glucose tolerance test according to the combined model of genetic variants (*GCK* and *INSR*) and chronic heavy alcohol consumption. *P* values for glucose levels at 0 min, 1 h, and 2 h post-OGTT were calculated by general linear models with adjustment for age, physical activity, smoking status, BMI, and follow-up period. Significant differences of means among genotype-alcohol consumption groups by Duncan test (a: highest mean; c: lowest mean; a > b > c).

To determine the factors responsible for these effects, the ß-cell function and the insulin sensitivity index were calculated by using the OGTTs. Participants who reported therapy with anti-diabetes medication were excluded in this analysis. At the end of follow-up, a significant difference in the fasting and 1 h post-OGTT glucose levels was observed between the chronic heavy drinkers and never-drinkers. Combined effects between alcohol consumption and genotype were apparent at 2 h post-OGTT and that levels were higher in chronic heavy drinkers who were carriers of the rs758989 C allele or non-carriers of the *INSR* AACT haplotype compared with never-drinkers carrying the C allele or AACT haplotype, respectively (Fig. 3 and Supplementary Fig. S4). Additionally, we observed that chronic heavy alcohol consumption was associated with decreased β-cell function, and decreasing late insulin secretion (I/G_120_ and I/G_AUC 60–120_) was particularly observed in chronic heavy drinkers carrying the *GCK* C allele compared with never-drinkers who were GCK C allele Table 3). The composite ISI and disposition index were significantly decreased in AACT haplotype non-carriers than carriers among the chronic heavy drinkers.

**Table 3:** Changes in ß-cell function and the insulin sensitivity index in the oral glucose tolerance test according to genetic variants in *GCK* and *INSR* and alcohol consumption patterns.

*GCK*	Never-drinker		*P*_0_^a^	Chronic heavy drinker		*P*_0_^a^	*P*_1_^b^	Post-hoc anlaysis
	TT	TC + CC		TT	TC + CC			
HOMA-B (%)	102.8 ± 51.7	105.2 ± 79.9	0.7956	90 ± 73.8	85.4 ± 67.2	0.4355	**<0.0001**	a,a,b,b
IGI_60_	11.8 ± 13.4	11.0 ± 13.0	0.5220	9.1 ± 12.2	7.1 ± 9.5	0.1191	**0.0002**	a,a,b,b
I/G_AUC 60–120_	4.4 ± 3.1	4.6 ± 3.4	0.6289	4.1 ± 3.3	3.4 ± 2.6	0.0523	**0.0004**	ab,a,b,c
I/G_120_	3.2 ± 2.8	3.8 ± 3.8	0.0116	3.0 ± 4.3	3.3 ± 4.7	0.6387	**0.0043**	b,a,b,b
Composite ISI	9.6 ± 4.8	10 0.0 ± 5.2	0.8024	9.8 ± 4.6	9.8 ± 5.1	0.9457	0.4124	
Disposition index	105.9 ± 137.3	105.2 ± 127.5	0.5390	76.6 ± 102.4	61.2 ± 85.1	0.076	**0.0023**	a,a,b,b
***INSR***	**AACT carrier**	**Non-carrier**		**AACT carrier**	**Non-carrier**			
HOMA-B (%)	105.4 ± 74.7	104.8 ± 57.3	0.8958	87.7 ± 81	89 ± 58.7	0.3553	**<0.0001**	a,a,b,b
IGI_60_	10.5 ± 11.7	12.2 ± 14.6	0.1874	8.4 ± 11	7.9 ± 11.5	0.1786	**0.0005**	ab,a,bc,c
I/G_AUC 60–120_	4.4 ± 3.1	4.6 ± 3.4	0.4561	4.1 ± 3.3	3.4 ± 2.6	0.9352	**0.004**	a,a,b,b
I/G_120_	3.3 ± 2.9	3.8 ± 3.8	0.3259	3.1 ± 4.6	3.2 ± 4.5	0.7709	**0.0574**	
Composite ISI	9.9 ± 5.2	9.5 ± 4.9	0.7062	10.3 ± 4.9	9.0 ± 4.4	0.0190	**0.0496**	ab,ab,a,b
Disposition index	101.2 ± 129	104.7 ± 135.4	0.6418	76.8 ± 99.8	57.1 ± 87.9	0.0131	**0.0011**	a,a,a,b

Data are expressed as the means ± SD and variables were log-transformed prior to analysis; HOMA-B, homeostasis model assessment-beta; IGI_60_, insulinogenic index at 1 hour post-OGTT; I/G_AUC 60–120,_ the ratio of area under the insulin curve to area under the glucose curve from 1 h to 2 h; I/G_120_, the ratio of insulin to glucose at 2 hour post-OGTT; ISI, insulin sensitivity index.

^a^Differences between the genotype groups (TT vs TC + CC for *GCK*, AACT carrier vs Non-carrier for *INSR*) were assessed by general linear models with adjustment for age, physical activity, smoking status, BMI, AST and tracking period.

^b^Differences among the genotype-alcohol consumption groups were assessed by general linear models with adjustment for age, physical activity, smoking status, BMI, AST and tracking period. Duncan post-hoc test was used to identify group differences (a: highest mean; c: lowest mean; a > b > c).

## Discussion

This is the first prospective study to investigate the effect of the interaction between genetic variants of *GCK*/*INSR* and chronic heavy alcohol consumption on the incidence of diabetes, using data from a 12-year follow-up cohort study designed to assess genetic and environmental risk factors for diabetes. In this study, we found that chronic heavy alcohol consumption was associated with the risk of developing diabetes in carriers of the *GCK* rs758989 C allele and in non-carriers of the *INSR* AACT haplotype.

The strength of our study is that we used estimates of β-cell function and insulin sensitivity derived from the OGTT, which provide more information about the dynamic responses of glucose and insulin than do basal steady state measurements^3^. In particular, previous study reported that measures of insulin secretion derived from the early and late OGTT periods were independent predictors of diabetes^13^.

In this study, we found that chronic heavy alcohol consumption was associated with a decrease in β-cell function, and with the development of diabetes. These results were consistent with those from previous studies, which reported that excess alcohol consumption had a deleterious effect on β-cell function by decreasing insulin secretion^7,14–16^ and exhibited an association with diabetes^16,17^. As a potential mechanism for this effect, ethanol induces endoplasmic reticulum stress and oxidative stress, which represent the earliest events in glucose intolerance and lead to pancreatic β-cell dysfunction, apoptosis, and eventually diabetes^15,18^. Several studies have reported that low-to-moderate alcohol consumption has a protective effect against diabetes by increasing insulin sensitivity^7,19^, but we did not find a significance in the difference of insulin sensitivity according to the alcohol consumption (Supplementary Table S7). A meta-analysis of 2–12-week intervention studies showed that alcohol consumption improved insulin sensitivity in women, but not in men^20^. Further studies conducted according to different variables, including sex and the alcohol exposure amount and period, are needed to understand the aspects of glucose metabolism that affect alcohol consumption.

Since chronic alcohol consumption can affect gene expression and alterations, comprehensive assessment of the effects of alcohol consumption–gene interactions on the development of diabetes is needed. In this study, we found that the association between a *GCK* rs758989 and diabetes development was affected by chronic heavy alcohol consumption. In addition, chronic heavy alcohol consumption was associated with a risk of developing diabetes in carriers of the rs758989 C allele but not the homozygous TT genotype. Moreover, carriers of the C allele had lower β-cell function and a higher incidence of diabetes among the chronic heavy drinkers than the never-drinkers. *GCK* plays a critical role as a β-cell glucose sensor by integrating glucose metabolism and insulin secretion^5^, and genetic variants of *GCK* have been associated with ß-cell function and diabetes development^3,21^. Previous studies reported that *GCK* expression was decreased in mice fed chronic ethanol^15^, and mice lacking hepatic *GCK* expression showed a type 2 diabetes phenotype at a young age^22^. These findings demonstrate that chronic alcohol consumption may increase pancreatic β-cell apoptosis and dysfunction via downregulation of *GCK* expression, resulting in development of diabetes^15^.

Similarly, defects in glucose intolerance have also been observed in mice lacking a functional *INSR* gene^23^. *INSR* is an important mediator between the extracellular and intracellular insulin signaling pathways, which phosphorylate IRS to activate downstream molecules, including PI3K pathway members. Mutations in *INSR* have been detected in individuals with extreme insulin resistance^24^ and confer a risk of non-insulin-dependent (type 2) diabetes mellitus^4,25,26^. The mechanisms have not been fully elucidated, but a few studies have suggested that ethanol inhibits *INSR* or the associations of *INSR* and *IRS-1* with the p85 subunit of PI3K, which is associated with insulin resistance^27,28^. In this study, we found that chronic heavy alcohol consumption influenced the associations between *INSR* variants and the incidence of diabetes. Chronic heavy drinkers who were non-carriers of the *INSR* AACT haplotype had a higher incidence of diabetes, indicated by decreased disposition index reflecting β-cell function and composite insulin sensitivity, compared with carriers of the AACT haplotype or never-drinkers. Chronic heavy alcohol consumption was associated with low insulin sensitivity in *INSR* AACT non-carriers, whereas a protective effect was observed in *INSR* AACT carriers. A previous longitudinal study in Korea reported low β-cell function during the early stage of diabetes development and a pronounced decrease in insulin sensitivity just before diabetes onset^3^. These findings may explain the low incidence of diabetes, characterized by decreased β-cell function and elevated insulin sensitivity, in carriers of the AACT haplotype compared with non-carriers among chronic heavy drinkers.

Another finding of this research was the effects of genotype and alcohol consumption on glucose level patterns during the OGTT. Chronic heavy drinkers had increased fasting and 1 h-OGTT glucose levels and decreased HOMA-B compared with never-drinkers. However, at 2 h, never-drinkers carrying the *GCK* TT homozygous showed similar levels of glucose as chronic heavy drinkers with TT genotype by decreasing their late insulin secretion. The 2 h post-OGTT glucose levels of chronic heavy drinkers with *INSR* AACT haplotype were recovered similar to never-drinkers by increasing their composite ISI. These results suggest that the 1 h post-OGTT glucose level is greatly influenced by alcohol consumption, and the 2 h post-OGTT glucose level is affected by the interaction between genotype and alcohol consumption. Although further studies are needed to validate this finding, it may help clarify the potential contribution of the interaction between genotype and alcohol consumption to diabetes risk.

Our study had several strengths. First, to our knowledge, this is the first investigation of the effects of the interactions between *GCK*/*INSR* genetic variants and chronic alcohol consumption on the incidence of diabetes using 12-year follow-up data, providing a detailed analysis based on glycemic index data obtained from OGTTs. Second, we used alcohol consumption data throughout the entire tracking period to reduce the bias introduced by measurements at a single time point. However, our study also had limitations, including the potential bias associated with the decision to respond, or not, to the alcohol survey. The never-drinkers had a similar incidence of diabetes as subjects who had chronic low to moderate or irregular drinking pattern (Supplementary Table S3). We assume that this is because individuals with poor health may be more likely not to drink. Further studies are needed to validate our findings. Also, we used IGI_60_ as the index of early insulin secretion because 30 min glucose and insulin values were not available. However, the index at 1 h correlates well with IGI_30_ and can be used as surrogate of early insulin secretion^10^.

Previously, we had reported that alcohol consumption affects insulin secretions^16,29^ and diabetes^16^. We emphasized that β-cell dysfunction caused by heavy alcohol consumption is associated with the development of diabetes in the follow-up study. In the present study, we investigated common genetic variants that interact with chronic heavy alcohol consumption affecting β-cell dysfunction and development of diabetes. For the main analysis, we excluded the low to moderate drinking group because there is a controversy that low to moderate drinking has the beneficial effect on incident diabetes. We did not observe the protective effect of low to moderate drinking on diabetes, which might be due to the heterogeneity in handling of missing values for diabetes and criteria for alcohol consumption pattern groups. In the analyses of the previous study^16^, we excluded the subjects who had more than one missing values in the information used to diagnosis diabetes, and whose response rate to the alcohol questionnaire was below 80% during the follow-up period. To identify the effects of low to moderated drinking on development of diabetes, the study should be designed well and presented clearly the criteria of analysis for the less confusion.

In summary, we showed an effect of the interactions between genetic variants and alcohol consumption on the incidence of diabetes. According to these results, chronic heavy alcohol consumption was associated with a risk of developing diabetes in carriers of the *GCK* C allele or non-carriers of the *INSR* AACT haplotype. We also found that alcohol consumption was associated with an increased 1 h post-OGTT glucose level and decreased β-cell function. Furthermore, the interaction between genotype and alcohol consumption was associated with the 2 h post-OGTT glucose level, late insulin secretion (*GCK* allele), and β-cell function reflecting insulin sensitivity (*INSR* haplotype). These results potentially suggest that chronic heavy alcohol consumption induce β-cell dysfunction partially mediated by decreased *GCK* expression or decline of insulin sensitivity via inhibition of *INSR*, thereby contributing to the development of diabetes.

## Supplementary information

Chronic heavy alcohol consumption influences the association between genetic variants of GCK or INSR and the development of diabetes in men: A 12-year follow-up study
