# Supplementary material for: Chronic heavy alcohol consumption influences the association between genetic variants of GCK or INSR and the development of diabetes in men: A 12-year follow-up study
Source: Sci Rep. 2019 Dec 27;9:20029. doi: 10.1038/s41598-019-56011-y (PMC6934767; doi:10.1038/s41598-019-56011-y)
Supplement: Supplementary file 1 — Chronic heavy alcohol consumption influences the association between genetic variants of GCK or INSR and the development of diabetes in men: A 12-year follow-up study [file 41598_2019_56011_MOESM1_ESM.docx]

**Chronic heavy alcohol consumption influences the association between genetic variants of *GCK* or *INSR* and the development of diabetes in men: A 12-year follow-up study**

Han Byul Jang^1^, Min Jin Go^2^, Sang Ick Park^1^, Hye-Ja Lee^1*^, Sung Beom Cho^2*^

^1^Center for Biomedical Science, Korea National Institute of Health, Cheongju, Chungcheongbuk-do, Republic of Korea

^2^Center for Genome Science, Korea National Institute of Health, Cheongju, Chungcheongbuk-do, Republic of Korea

^*^Address for correspondence

Hye-Ja Lee, PhD

Center for Biomedical Science, Korea National Institute of Health

Tel. 82-043-719-8692, Fax. 82-043-719-8602, Email. [hyejalee@yahoo.co.kr](mailto:hyejalee@yahoo.co.kr)

Sung Beom Cho, PhD

Center for Genome Science, Korea National Institute of Health

Tel. 82-043-719-8850, Fax. 82-043-719-8602, Email. [sbcho@korea.kr](mailto:sbcho@korea.kr)

Supplementary Tables

Supplementary Table S1. Characteristics of the subjects according to baseline alcohol consumption.

|  | Abstainers (N=726) | | Heavy drinkers (N=794) | | *P*-value ^b^ |
| --- | --- | --- | --- | --- | --- |
| Age (y) | 52.5 | ± 8.9 | 50.0 | ± 8.5 | <0.0001 |
| BMI (kg/m^2^) | 24.0 | ± 3.0 | 24.3 | ± 3.0 | 0.2306 |
| SBP (mmHg) | 119.9 | ± 15.6 | 124.2 | ± 17.5 | <.0001 |
| DBP (mmHg) | 80.0 | ± 10.2 | 83.8 | ± 11.6 | <.0001 |
| AST (IU/L)^a^ | 26.4 | ± 14.7 | 33.6 | ± 23.4 | <.0001 |
| ALT (IU/L)^a^ | 28.3 | ± 20.0 | 31.8 | ± 21.6 | 0.0002 |
| GTP (IU/L)^a^ | 30.5 | ± 32.6 | 89.8 | ± 133.1 | <.0001 |
| Total cholesterol (mg/dL) | 5.1 | ± 0.9 | 5.1 | ± 1.0 | 0.7707 |
| HDL-cholesterol (mg/dL) | 1.1 | ± 0.2 | 1.3 | ± 0.3 | <.0001 |
| Triglycerides (mg/dL)^a^ | 1.7 | ± 1.1 | 2.2 | ± 1.7 | <.0001 |
| Fasting glucose | 5.0 | ± 1.0 | 5.4 | ± 1.5 | <.0001 |
| 1 h glucose | 9.0 | ± 3.3 | 9.9 | ± 3.7 | <.0001 |
| 2 h glucose | 7.1 | ± 3.1 | 7.8 | ± 3.8 | <.0001 |
| Fasting insulin (μU /mL)^a^ | 7.1 | ± 5.2 | 6.7 | ± 3.3 | 0.0565 |
| 1 h insulin (μU /mL)^a^ | 30.8 | ± 32.7 | 28.4 | ± 27.6 | 0.0256 |
| 2 h insulin (μU /mL)^a^ | 25.5 | ± 24.4 | 23.5 | ± 24.4 | 0.0073 |
| HOMA-B (%)^a^ | 108.8 | ± 93.3 | 85.0 | ± 56.5 | <.0001 |
| IGI _60_^a^ | 9.0 | ± 14.7 | 7.2 | ± 13.2 | 0.0002 |
| I/G _AUC_ _60-120_^a^ | 3.3 | ± 2.6 | 2.8 | ± 2.4 | <.0001 |
| I/G _120_^a^ | 3.7 | ± 3.4 | 3.2 | ± 3.0 | <.0001 |
| Composite ISI^a^ | 11.8 | ± 10.8 | 11.8 | ± 9.7 | 0.5760 |
| Disposition index^a^ | 72.4 | ± 91.8 | 59.1 | ± 78.4 | 0.0011 |
| MET (Physical activity) | 9952.7 | ± 6364.8 | 10351.8 | ± 6391.4 | 0.0078 |
| Smoking (Ex/Current) | 27.3% | /37.4% | 28.0% | /61.1% | <.0001 |
| Family history of diabetes | 9.8% | | 11.1% | | 0.4069 |
| Prevalence of prediabetes | 23.4% | | 30.6% | | <.0001 |
| diabetes | 9.2% | | 16.0% | |  |

Data are unadjusted means (SD) or %; BMI, body mass index; SBP, systolic blood pressure; DBP, diastolic blood pressure; ALT, alanine aminotransferase; AST, aspartate aminotransferase; r-GTP, gamma glutamyltranspeptidase; HOMA-B, homeostasis model assessment-beta; IGI, insulinogenic index; I/G, the ratio of insulin to glucose; AUC, area under the curve; ISI, insulin sensitivity index; MET, metabolic equivalent of task; ^a^ Log transformations before analysis; ^b^ *P* value were calculated by generalized linear regression analysis with age for continuous parametric variables and Chi-square test for categorical variables.

Supplementary Table S2. Cross-sectional analysis of the role of interactions between *GCK* or *INSR* single-nucleotide polymorphisms and alcohol consumption in prediabetes and diabetes.

| **CHR** | **BP** | **SNP** | **Region** | **MAF** | **Allele** | **Prediabetes + Diabetes** | | | | | | | | |
| --- | --- | --- | --- | --- | --- | --- | --- | --- | --- | --- | --- | --- | --- | --- |
|  |  |  |  |  |  | **Gene** | |  | **Drinking** | |  | **Gene*Drinking** | |  |
| **GCK** |  |  |  |  |  |  |  |  |  |  |  |  |  |  |
| 7 | 44,166,624 | rs17832252 | intron Upstream(5000bp) | 0.23 | T<G | 1.59 | 0.112 |  | 2.04 | **1.11E-07** | **^*^** | 0.76 | 0.1225 |  |
| 7 | 44,169,531 | rs758989 | intron Upstream(5000bp) | 0.28 | C<T | 0.43 | **0.003** | **^*^** | 1.32 | **4.82E-02** |  | 1.79 | **0.0006** | **^*^** |
| 7 | 44,185,381 | rs2300586 | intron | 0.37 | T<C | 1.13 | 0.616 |  | 2.00 | **1.43E-05** | **^*^** | 0.88 | 0.4204 |  |
| 7 | 44,195,593 | rs1799884 | Upstream(5000bp) | 0.19 | A<G | 0.74 | 0.333 |  | 1.61 | **2.44E-04** | **^*^** | 1.35 | 0.1133 |  |
| **INSR** |  |  |  |  |  |  |  |  |  |  |  |  |  |  |
| 19 | 7,091,525 | rs4804304 | intron | 0.03 | G<A | 1.73 | 0.428 |  | 1.83 | **3.97E-08** | **^*^** | 0.84 | 0.6795 |  |
| 19 | 7,092,775 | rs2229431 | nonsynonymous | 0.10 | T<C | 0.73 | 0.489 |  | 1.82 | **3.16E-07** | **^*^** | 1.01 | 0.9767 |  |
| 19 | 7,093,843 | rs2229430 | nonsynonymous | 0.03 | C<G | 1.92 | 0.340 |  | 1.83 | **4.04E-08** | **^*^** | 0.79 | 0.5902 |  |
| 19 | 7,093,921 | rs2229434 | nonsynonymous | 0.03 | A<G | 1.92 | 0.340 |  | 1.83 | **4.04E-08** | **^*^** | 0.79 | 0.5902 |  |
| 19 | 7,114,154 | rs2963 | nonsynonymous | 0.02 | A<G | 0.88 | 0.874 |  | 1.81 | **6.78E-08** | **^*^** | 1.07 | 0.8806 |  |
| 19 | 7,117,109 | rs2059807 | intron | 0.28 | C<T | 1.18 | 0.552 |  | 1.88 | **1.01E-05** | **^*^** | 0.93 | 0.6659 |  |
| 19 | 7,117,138 | rs3815902 | intron | 0.20 | A<G | 1.03 | 0.927 |  | 1.79 | **7.65E-06** | **^*^** | 1.01 | 0.9669 |  |
| 19 | 7,121,882 | rs16994298 | intron | 0.02 | A<G | 3.68 | 0.137 |  | 1.85 | **1.30E-08** | **^*^** | 0.50 | 0.2457 |  |
| 19 | 7,122,629 | rs8109559 | intron | 0.04 | A<G | 1.17 | 0.791 |  | 1.82 | **7.60E-08** | **^*^** | 0.88 | 0.7281 |  |
| 19 | 7,133,753 | rs8108622 | intron | 0.14 | T<A | 1.43 | 0.300 |  | 1.86 | **5.42E-07** | **^*^** | 0.92 | 0.6981 |  |
| 19 | 7,133,963 | rs10500204 | intron | 0.15 | C<A | 1.33 | 0.411 |  | 1.81 | **1.83E-06** | **^*^** | 0.96 | 0.8479 |  |
| 19 | 7,138,628 | rs7245757 | intron | 0.34 | A<G | 1.57 | 0.088 |  | 2.28 | **2.16E-07** |  | 0.71 | **0.0366** |  |
| 19 | 7,150,803 | rs1035942 | intron | 0.33 | A<G | 1.87 | **0.015** |  | 2.42 | **6.53E-09** | **^*^** | 0.64 | **0.0058** |  |
| 19 | 7,150,978 | rs1035940 | intron | 0.34 | C<G | 1.81 | **0.017** |  | 2.37 | **1.27E-08** | **^*^** | 0.67 | **0.0100** |  |
| 19 | 7,155,394 | rs2042901 | intron | 0.33 | T<G | 1.88 | **0.014** |  | 2.42 | **6.33E-09** | **^*^** | 0.64 | **0.0060** |  |
| 19 | 7,162,816 | rs3745546 | intron | 0.13 | G<C | 1.14 | 0.716 |  | 1.79 | **1.95E-06** | **^*^** | 1.03 | 0.9061 |  |
| 19 | 7,162,841 | rs3745545 | intron | 0.06 | C<T | 1.15 | 0.782 |  | 1.82 | **1.39E-07** | **^*^** | 0.93 | 0.8125 |  |
| 19 | 7,169,135 | rs7245562 | intron | 0.29 | A<G | 1.55 | 0.100 |  | 2.16 | **7.02E-08** | **^*^** | 0.73 | 0.0556 |  |
| 19 | 7,173,832 | rs7508679 | intron | 0.44 | G<A | 0.74 | 0.224 |  | 1.57 | **8.27E-03** | **^*^** | 1.18 | 0.2663 |  |
| 19 | 7,181,438 | rs10416429 | intron | 0.19 | A<C | 0.93 | 0.809 |  | 1.80 | **4.18E-06** | **^*^** | 1.00 | 0.9889 |  |
| 19 | 7,184,604 | rs890862 | intron | 0.19 | T<C | 1.03 | 0.931 |  | 1.83 | **2.56E-06** | **^*^** | 0.95 | 0.7761 |  |
| 19 | 7,212,441 | rs919275 | intron | 0.26 | G<A | 0.74 | 0.280 |  | 1.65 | **2.83E-04** | **^*^** | 1.19 | 0.3146 |  |
| 19 | 7,244,119 | rs8101064 | intron | 0.13 | T<C | 1.05 | 0.890 |  | 1.87 | **2.41E-07** | **^*^** | 0.86 | 0.5030 |  |

^*^ Significant differences (P < 0.05) in genotype-environment interaction analysis for diabetes

Supplementary Table S3. Incidence of diabetes according to drinking patterns over the follow-up period.

A. Incidence of diabetes (%) according to alcohol consumption patterns.

| Class | Incident diabetes |
| --- | --- |
| Never-drinkers | 18.7 % |
| Chronic low to moderate drinkers | 19.8 % |
| Chronic heavy drinkers | 26.2 % |
| The rest of the participants (Irregular drinking pattern) | 18.8 % |

B. Hazard ratio for the incidence of diabetes in heavy drinkers compared with non-drinkers.

|  | Never-drinkers | | Chronic heavy drinkers | | *P*-value |
| --- | --- | --- | --- | --- | --- |
| Diabetes | HR | (95% CI) | HR | (95% CI) |  |
| Model1 | 1 | (reference) | 1.52 | (1.09-2.13) | 0.0138 |
| Model2 | 1 | (reference) | 1.50 | (1.07-2.01) | 0.0189 |
| Model3 | 1 | (reference) | 1.41 | (1.00-1.98) | 0.0479 |

HR, Hazard Ratio; CI, Confidence Interval

Values are HR (95% CI) for chronic heavy drinkers determined using a Cox proportional hazard model; Model 1, adjusted for age, physical activity, family history of diabetes, and smoking status; Model 2, adjusted for Model 1 + baseline ALT; Model 3, adjusted for Model 2 + baseline BMI

Supplementary Table S4. Baseline characteristics of the study participants according to rs758989 (GCK) in the longitudinal study.

|  | TT (N=421) | | TC (N=296) | | CC (N=56) | | *P*-value ^b^ |
| --- | --- | --- | --- | --- | --- | --- | --- |
|  | Mean | ± SD | Mean | ± SD | Mean | ± SD |  |
| Age (y) | 51.8 | ± 9.1 | 50.4 | ± 8.5 | 51.7 | ± 9.2 | 0.1247 |
| BMI (kg/m^2^) | 24.1 | ± 3.1 | 23.9 | ± 2.8 | 24.2 | ± 3.3 | 0.3455 |
| SBP (mmHg) | 122.3 | ± 16.8 | 120.2 | ± 15.1 | 123.9 | ± 16.5 | 0.2781 |
| DBP (mmHg) | 82.1 | ± 11.2 | 81.1 | ± 10.6 | 84.0 | ± 9.6 | 0.1720 |
| AST (IU/L)^a^ | 30.3 | ± 21.5 | 30.1 | ± 19.8 | 31.9 | ± 22.0 | 0.6862 |
| ALT (IU/L)^a^ | 29.5 | ± 20.6 | 30.0 | ± 19.7 | 30.9 | ± 18.5 | 0.5680 |
| GTP (IU/L)^a^ | 58.1 | ± 106.6 | 56.7 | ± 72.3 | 70.8 | ± 76.1 | 0.5282 |
| Total cholesterol (mmol/L) | 5.1 | ± 0.9 | 5.1 | ± 0.9 | 5.2 | ± 1.0 | 0.3864 |
| HDL-cholesterol (mmol/L) | 1.2 | ± 0.3 | 1.3 | ± 0.3 | 1.3 | ± 0.3 | 0.6621 |
| Triglycerides (mmol/L)^a^ | 1.9 | ± 1.5 | 2.0 | ± 1.5 | 2.3 | ± 2.2 | 0.2522 |
| Fasting glucose (mmol/L) | 5.0 | ± 0.5 | 5.0 | ± 0.5 | 5.0 | ± 0.6 | 0.1432 |
| 1 h glucose (mmol/L) | 8.8 | ± 2.4 | 8.8 | ± 2.7 | 9.3 | ± 2.7 | 0.3596 |
| 2 h glucose (mmol/L) | 6.5 | ± 1.9 | 6.5 | ± 1.9 | 7.2 | ± 1.7 | 0.0462 |
| Fasting insulin (μU /mL)^a^ | 6.6 | ± 3.5 | 7.1 | ± 5.9 | 6.6 | ± 2.8 | 0.5943 |
| 1 h insulin (μU /mL)^a^ | 31.0 | ± 29.4 | 28.5 | ± 29.5 | 27.5 | ± 23.1 | 0.6225 |
| 2 h insulin (μU /mL)^a^ | 22.5 | ± 22.4 | 25.2 | ± 26.7 | 25.8 | ± 20.1 | 0.2189 |
| HOMA-B (%)^a^ | 100.7 | ± 61.3 | 102.3 | ± 112.5 | 95.2 | ± 51.6 | 0.9902 |
| IGI _60_^a^ | 8.6 | ± 14.2 | 9.9 | ± 18.9 | 5.8 | ± 6.1 | 0.9645 |
| I/G _AUC_ _60-120_^a^ | 3.2 | ± 2.5 | 3.1 | ± 2.5 | 2.8 | ± 1.8 | 0.7923 |
| I/G _120_^a^ | 3.4 | ± 2.9 | 3.7 | ± 3.3 | 3.5 | ± 2.3 | 0.4647 |
| Composite ISI^a^ | 12.9 | ± 11.9 | 12.5 | ± 10.3 | 11.8 | ± 10.6 | 0.6590 |
| Disposition index^a^ | 70.1 | ± 80.9 | 73.9 | ± 104.9 | 62.2 | ± 76.5 | 0.8520 |
| MET (Physical activity) | 10570 | ± 6466.1 | 10729 | ± 6427.9 | 10147 | ± 6754 | 0.5705 |
| Smoking (Ex/Current) | 29.3 | /47.9% | 24.0 | /55.4% | 30.4 | /52.6% | 0.2650 |
| Family history of diabetes | 11.2% | | 11.5% | | 8.9% | | 0.8551 |
| Incident of diabetes | 21.9% | | 23.3% | | 26.8% | | 0.6824 |

Data are unadjusted means (SD) or %; BMI, body mass index; SBP, systolic blood pressure; DBP, diastolic blood pressure; ALT, alanine aminotransferase; AST, aspartate aminotransferase; r-GTP, gamma glutamyltranspeptidase; HOMA-B, homeostasis model assessment-beta; IGI, insulinogenic index; I/G, the ratio of insulin to glucose; AUC, area under the curve; ISI, insulin sensitivity index; MET, metabolic equivalent of task; ^a^ Log transformations before analysis; ^b^ *P* value were calculated by generalized linear regression analysis with age for continuous parametric variables and Chi-square test for categorical variables.

Supplementary Table S5. Baseline characteristics of the study participants according to *INSR* haplotype in the longitudinal study.

|  | Non-carriers (N=327) | | AACT Carriers (N=403) | | *P*-value ^b^ |
| --- | --- | --- | --- | --- | --- |
| Age (y) | 51.5 | ± 8.8 | 51.1 | ± 8.9 | 0.5599 |
| BMI (kg/m^2^) | 24.3 | ± 3.0 | 23.9 | ± 3.0 | 0.0627 |
| SBP (mmHg) | 122.7 | ± 17.0 | 120.8 | ± 15.5 | 0.1446 |
| DBP (mmHg) | 82.8 | ± 11.4 | 81.3 | ± 10.5 | 0.0628 |
| AST (IU/L) ^a^ | 29.9 | ± 19.1 | 31.0 | ± 22.8 | 0.7265 |
| ALT (IU/L) ^a^ | 29.7 | ± 18.4 | 29.9 | ± 21.7 | 0.4836 |
| GTP (IU/L) ^a^ | 56.5 | ± 86.6 | 61.8 | ± 101.5 | 0.4891 |
| Total cholesterol (mmol/L) | 5.2 | ± 0.9 | 5.1 | ± 0.9 | 0.1014 |
| HDL-cholesterol (mmol/L) | 1.2 | ± 0.3 | 1.3 | ± 0.3 | 0.2418 |
| Triglycerides (mmol/L)^a^ | 2.0 | ± 1.5 | 1.9 | ± 1.6 | 0.1102 |
| Fasting glucose (mmol/L) | 5.1 | ± 0.5 | 4.9 | ± 0.5 | 0.0047 |
| 1 h glucose (mmol/L) | 9.1 | ± 2.4 | 8.7 | ± 2.6 | 0.0384 |
| 2 h glucose (mmol/L) | 6.9 | ± 1.9 | 6.3 | ± 1.8 | <.0001 |
| Fasting insulin (μU /mL)^a^ | 6.7 | ± 3.7 | 6.9 | ± 5.3 | 0.7402 |
| 1 h insulin (μU /mL)^a^ | 31.1 | ± 30.5 | 29.3 | ± 28.3 | 0.5235 |
| 2 h insulin (μU /mL)^a^ | 25.6 | ± 26.1 | 22.8 | ± 22.9 | 0.0235 |
| HOMA-B (%)^a^ | 94.2 | ± 55.8 | 106.9 | ± 104.1 | 0.0657 |
| IGI _60_ ^a^ | 8.0 | ± 12.1 | 9.8 | ± 18.4 | 0.4016 |
| I/G _AUC_ _60-120_ ^a^ | 3.6 | ± 2.9 | 3.5 | ± 2.9 | 0.6160 |
| I/G _120_^a^ | 3.7 | ± 3.2 | 3.4 | ± 2.9 | 0.2695 |
| Composite ISI ^a^ | 12.1 | ± 11.8 | 13.1 | ± 11.1 | 0.1235 |
| Disposition index ^a^ | 69.3 | ± 82.0 | 72.1 | ± 95.7 | 0.5361 |
| MET (Physical activity) | 10235.4 | ± 6404.3 | 10916.7 | ± 6524.7 | 0.1198 |
| Smoking (Ex/Current) | 30.1% | /47.2% | 25.3% | /53.6% | 0.2089 |
| Family history of diabetes | 11.6% | | 10.7% | | 0.6842 |
| Incident of diabetes | 27.8% | | 18.9% | | 0.0041 |

Data are unadjusted means (SD) or %; BMI, body mass index; SBP, systolic blood pressure; DBP, diastolic blood pressure; ALT, alanine aminotransferase; AST, aspartate aminotransferase; r-GTP, gamma glutamyltranspeptidase; HOMA-B, homeostasis model assessment-beta; IGI, insulinogenic index; I/G, the ratio of insulin to glucose; AUC, area under the curve; ISI, insulin sensitivity index; MET, metabolic equivalent of task; ^a^ Log transformations before analysis; ^b^ *P* value were calculated by generalized linear regression analysis with age for continuous parametric variables and Chi-square test for categorical variables.

Supplementary Table S6. Incidence of diabetes according to single-nucleotide polymorphisms in *GCK* and *INSR* and alcohol consumption patterns.

|  | CC^a^ | | | CR^a^ | | | RR^a^ | | | *p* for trend ^b^ |
| --- | --- | --- | --- | --- | --- | --- | --- | --- | --- | --- |
|  | DM/Total | HR | (CI) | DM/Total | HR | (CI) | DM/Total | HR | (CI) |  |
| ***GCK*** |  |  |  |  |  |  |  |  |  |  |
| rs758989 |  |  |  |  |  |  |  |  |  |  |
| All | 92/421 | 1 | (reference) | 69/296 | 1.04 | (0.76-1.44) | 15/56 | 1.25 | (0.71-2.20) | 0.5008 |
| Never-drinker | 42/195 | 1 | (reference) | 22/137 | 0.70 | (0.41-1.20) | 2/21 | 0.43 | (0.10-1.79) | 0.0994 |
| Chronic heavy drinker | 50/226 | 1 | (reference) | 47/159 | 1.41 | (0.94-2.13) | 13/35 | 1.68 | (0.89-3.18) | 0.0436 |
| ***INSR*** |  |  |  |  |  |  |  |  |  |  |
| rs7245757 |  |  |  |  |  |  |  |  |  |  |
| All | 87/319 | 2.27 | (1.17-4.37) | 70/337 | 1.81 | (0.93-3.55) | 10/76 | 1 | (reference) | 0.0104 |
| Never-drinker | 31/146 | 1.61 | (0.61-4.27) | 25/150 | 1.43 | (0.53-3.87) | 5/34 | 1 | (reference) | 0.3394 |
| Chronic heavy drinker | 56/173 | 3.20 | (1.27-8.02) | 45/187 | 2.50 | (0.98-6.37) | 5/42 | 1 | (reference) | 0.0103 |
| rs1035940 |  |  |  |  |  |  |  |  |  |  |
| All | 97/357 | 1.92 | (1.09-3.38) | 65/325 | 1.46 | (0.81-2.62) | 14/91 | 1 | (reference) | 0.0096 |
| Non-drinker | 34/164 | 1.33 | (0.58-3.07) | 25/150 | 1.21 | (0.51-2.88) | 7/39 | 1 | (reference) | 0.4930 |
| Chronic heavy drinker | 63/193 | 2.77 | (1.26-6.07) | 40/175 | 2.01 | (0.89-4.53) | 7/52 | 1 | (reference) | 0.0053 |
| rs1035942 |  |  |  |  |  |  |  |  |  |  |
| All | 97/357 | 1.91 | (1.04-3.49) | 26/157 | 1.43 | (0.77-2.66) | 12/77 | 1 | (reference) | 0.0118 |
| Never-drinker | 34/164 | 1.01 | (0.99-1.02) | 41/180 | 0.89 | (0.61-1.31) | 6/31 | 1 | (reference) | 0.5604 |
| Chronic heavy drinker | 63/193 | 2.87 | (1.23-6.66) |  | 2.06 | (0.87-4.90) | 6/46 | 1 | (reference) | 0.0059 |
| rs2042901 |  |  |  |  |  |  |  |  |  |  |
| All | 97/357 | 1.91 | (1.04-3.49) | 67/340 | 1.42 | (0.76-2.65) | 12/76 | 1 | (reference) | 0.0114 |
| Never-drinker | 34/164 | 1.24 | (0.50-3.06) | 26/158 | 1.10 | (0.43-2.79) | 6/31 | 1 | (reference) | 0.5562 |
| Chronic heavy drinker | 63/193 | 2.87 | (1.24-6.67) | 41/182 | 2.05 | (0.86-4.88) | 6/45 | 1 | (reference) | 0.0057 |

DM, diabetes; HR, Hazard Ratio; CI, Confidence Interval; ^a^ C, Common allele; R, Rare allele; ^b^ *P* value for trend of the additive models was calculated by logistic regression analysis with adjustment for age, physical activity, family history of diabetes, smoking status, BMI and ALT.

Supplementary Table S7. Changes in ß-cell function and the insulin sensitivity index in the oral glucose tolerance test according to alcohol consumption patterns ^a^.

|  | Never-drinkers | Chronic low to moderate drinkers | Chronic heavy drinkers | *P*-value |
| --- | --- | --- | --- | --- |
| HOMA-B (%) | 103.9 ± 65.7 | 95.2 ± 47.8 | 87.9 ± 70.7 | <.0001 |
| IGI _60_ | 11.5 ± 13.2 | 9.8 ± 11.9 | 8.2 ± 11.1 | <.0001 |
| I/G _AUC_ _1-2_ | 4.0 ± 2.7 | 3.8 ± 2.7 | 3.4 ± 2.6 | <.0001 |
| I/G _120_ | 3.8 ± 3.1 | 3.8 ± 3.6 | 3.3 ± 2.9 | 0.0956 |
| Composite ISI | 9.8 ± 5.0 | 9.5 ± 4.8 | 9.8 ± 4.8 | 0.1921 |
| Disposition index | 105.6 ± 132.8 | 81.1 ± 93.2 | 69.6 ± 95.1 | <.0001 |

Data are expressed as the means±SD and variables were log-transformed prior to analysis; HOMA-B, homeostasis model assessment-beta; IGI_60_, insulinogenic index at 1 hour post-OGTT; I/G _AUC_ _60-120,_ the ratio of area under the insulin curve to area under the glucose curve from 1 h to 2 h; I/G_120_, the ratio of insulin to glucose at 2 hour post-OGTT; ISI, insulin sensitivity index; *P* values are adjusted for age, physical activity, family history of diabetes, smoking status, BMI, ALT tracking period.

Supplementary Figures


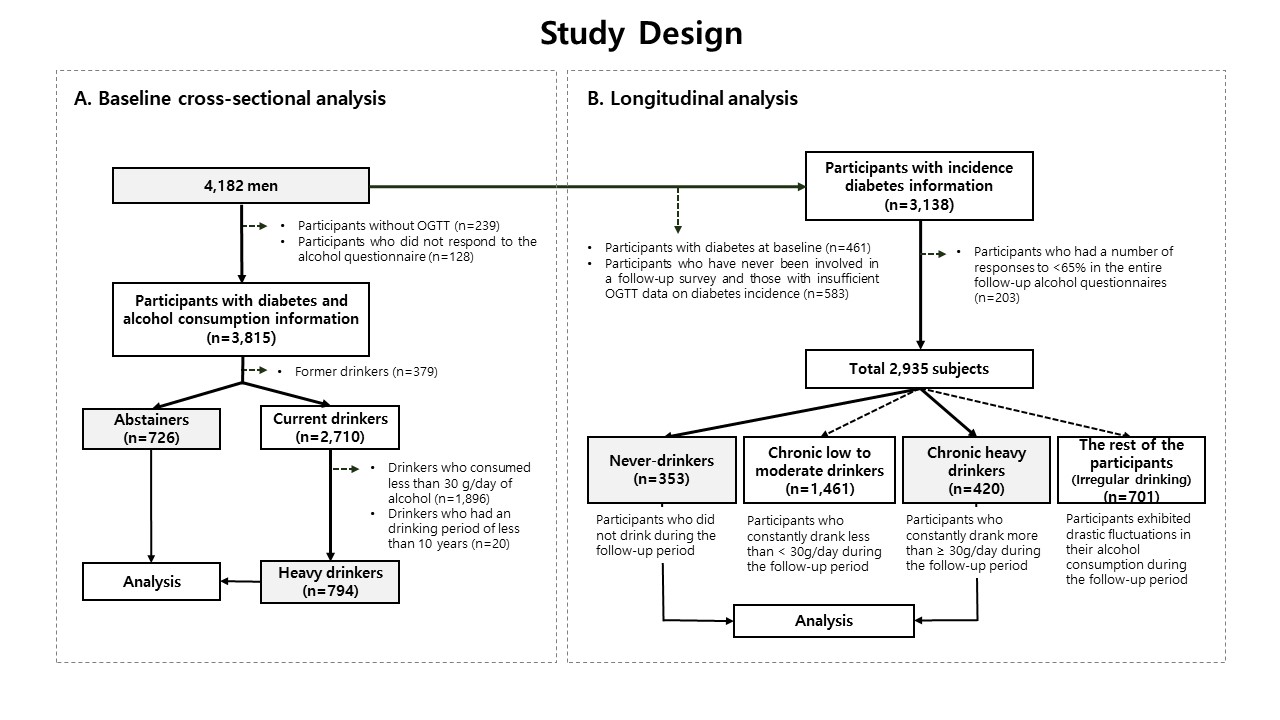


Supplementary Fig. S1. Flow Chart of the study design. (A) Cross-sectional analysis (B) Longitudinal analysis


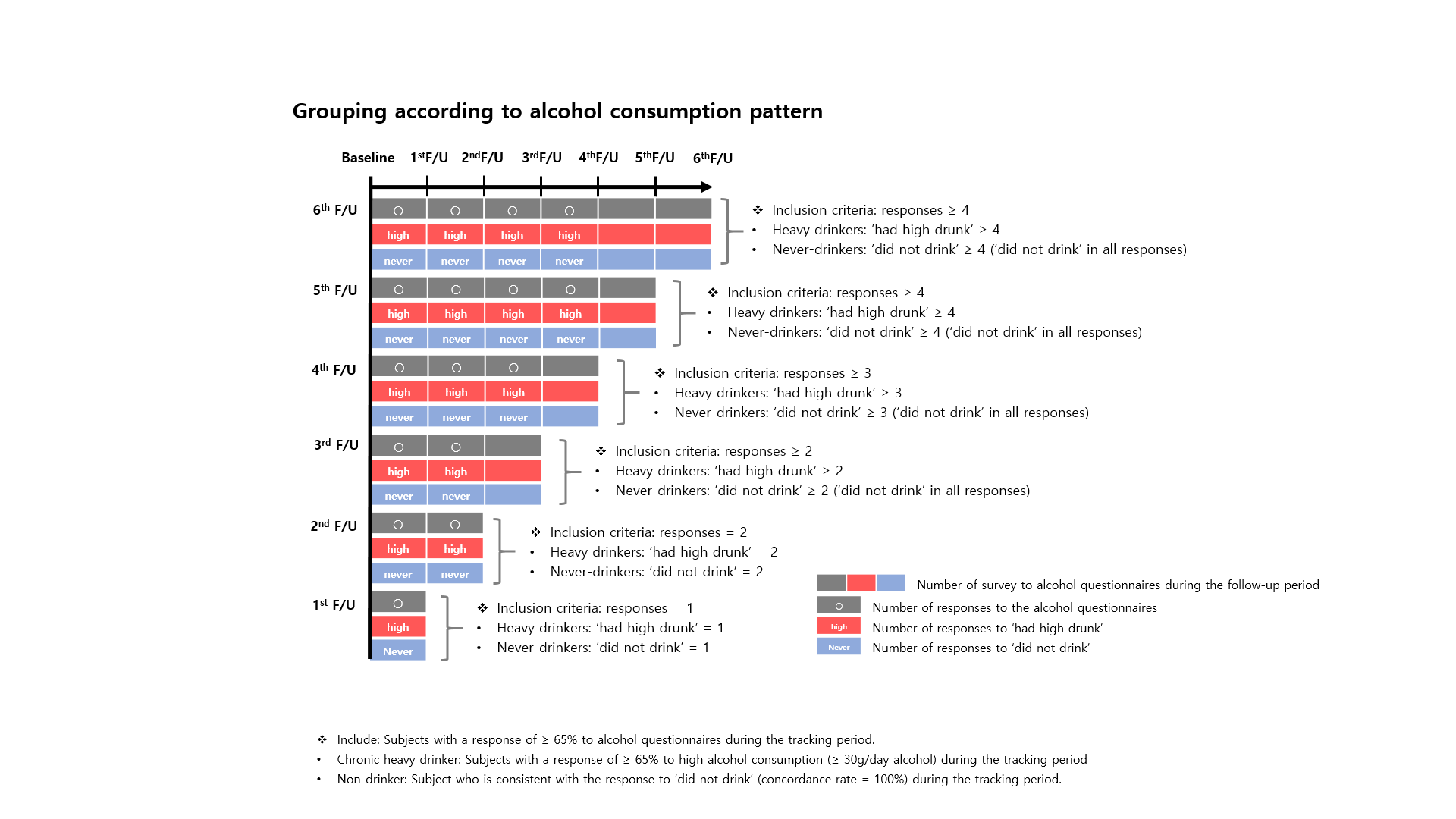


Supplementary Fig. S2. Grouping according the alcohol consumption pattern. Subjects with responses of ≥ 65% to alcohol questionnaires during the tracking period were included in this study. Subjects with high drinking (30g/day) rate of ≥ 65% during the tracking period were assigned to the chronic heavy drinkers. Subjects who were consistent with the response to ‘did not drink’ during the period (concordance rate = 100%) were assigned to never-drinkers.

Supplementary Fig. S3. Kaplan–Meier curves for the incidence of diabetes according to genetic variants of (A) additive model of *GCK* or (B) dominant of *GCK* or (C) *INSR* haplotype and (D) alcohol consumption pattern.


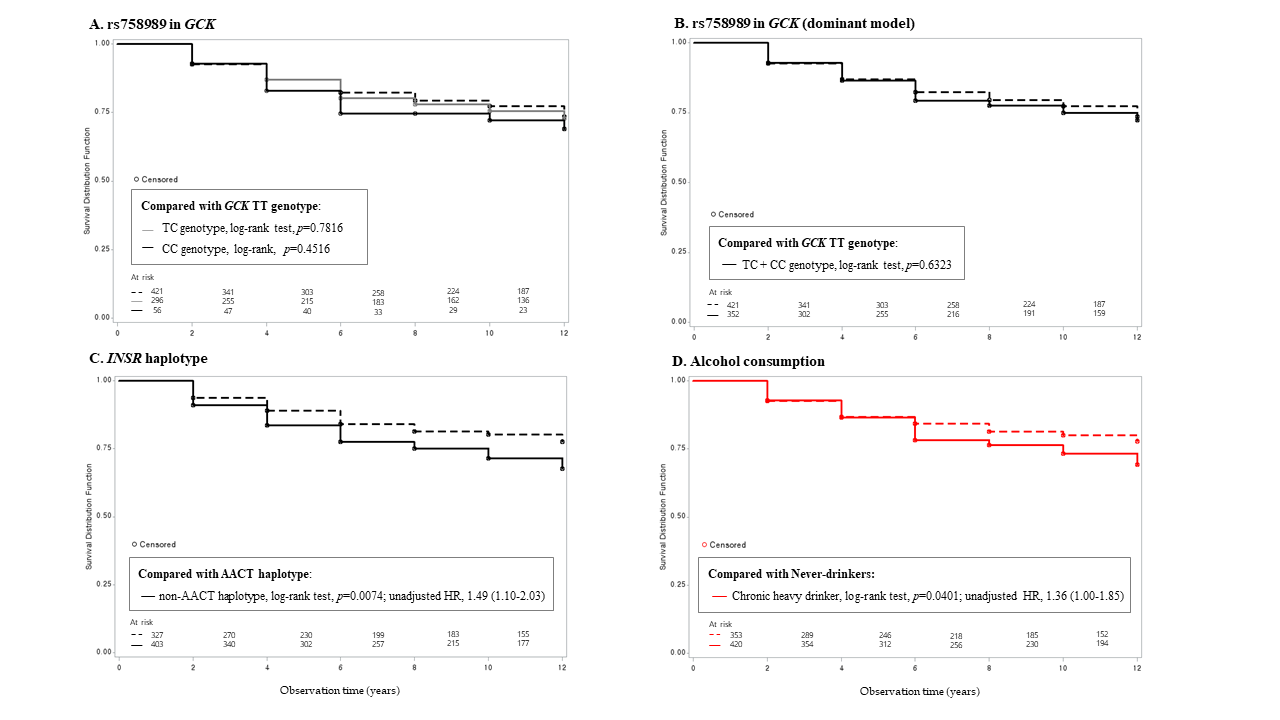


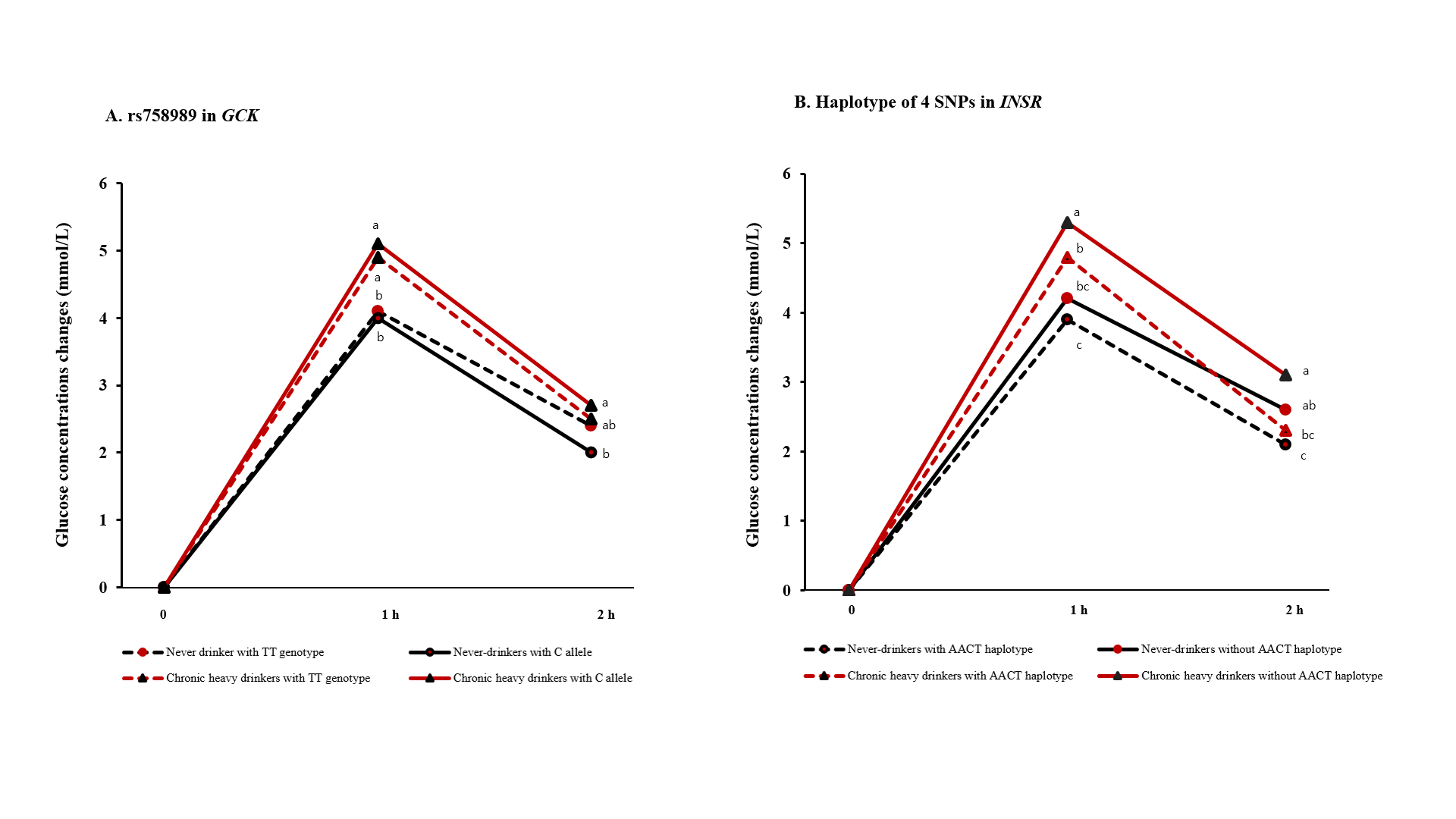


Supplementary Fig. S4. Changes in glucose concentrations during the oral glucose tolerance test according to the combined model of genetic variants (*GCK* and *INSR*) and chronic heavy alcohol consumption.
